# Molecular epidemiology of measles viruses in China, 1995–2003

**DOI:** 10.1186/1743-422X-4-14

**Published:** 2007-02-05

**Authors:** Yan Zhang, Zhen Zhu, Paul A Rota, Xiaohong Jiang, Jiayu Hu, Jianguo Wang, Wei Tang, Zhenying Zhang, Congyong Li, Changyin Wang, Tongzhan Wang, Lei Zheng, Hong Tian, Hua Ling, Chunfang Zhao, Yan Ma, Chunyan Lin, Jilan He, Jiang Tian, Yan Ma, Ping Li, Ronghui Guan, Weikuan He, Jianhui Zhou, Guiyan Liu, Hong Zhang, Xinge Yan, Xuelei Yang, Jinlin Zhang, Yiyu Lu, Shunde Zhou, Zhuoma Ba, Wei Liu, Xiuhui Yang, Yujie Ma, Yong Liang, Yeqiang Li, Yixin Ji, David Featherstone, William J Bellini, Songtao Xu, Guodong Liang, Wenbo Xu

**Affiliations:** 1 WHO Regional Reference Laboratory for Measles for the Western Pacific Region, National Institute for Viral Disease Control and Prevention, China Center for Disease Control and Prevention, Beijing 100050, China; 2 Division of Viral Diseases, Centers for Disease Control and Prevention, 1600 Clifton Road, Atlanta, GA 30333, USA; 3 Shanghai Provincial Center for Disease Control and Prevention, China; 4 Henan Provincial Center for Disease Control and Prevention, China; 5 Shandong Provincial Center for Disease Control and Prevention, China; 6 Shanxi Provincial Center for Disease Control and Prevention, China; 7 Tianjin Provincial Center for Disease Control and Prevention, China; 8 Chongqing Provincial Center for Disease Control and Prevention, China; 9 Hainan Provincial Center for Disease Control and Prevention, China; 10 Sichuan Provincial Center for Disease Control and Prevention, China; 11 Liaoning Provincial Center for Disease Control and Prevention, China; 12 Shannxi Provincial Center for Disease Control and Prevention, China; 13 Anhui Provincial Center for Disease Control and Prevention, China; 14 Jilin Provincial Center for Disease Control and Prevention, China; 15 Hunan Provincial Center for Disease Control and Prevention, China; 16 Guangdong Provincial Center for Disease Control and Prevention, China; 17 Xinjiang Provincial Center for Disease Control and Prevention, China; 18 Jiangsu Provincial Center for Disease Control and Prevention, China; 19 Zhejiang Provincial Center for Disease Control and Prevention, China; 20 Jiangxi Provincial Center for Disease Control and Prevention, China; 21 Qinghai Provincial Center for Disease Control and Prevention, China; 22 Jiangxi Provincial Center for Disease Control and Prevention, China; 23 Fujian Provincial Center for Disease Control and Prevention, China; 24 Heilongjiang Provincial Center for Disease Control and Prevention, China; 25 Hebei Provincial Center for Disease Control and Prevention, China; 26 Immunization, Vaccines and Biologicals, World Health Organization, Geneva, Switzerland; 27 National Institute for Viral Disease Control and Prevention, China Center for Disease Control and Prevention, Beijing 100052, China

## Abstract

This report describes the genetic characterization of 297 wild-type measles viruses that were isolated in 24 provinces of China between 1995 and 2003. Phylogenetic analysis of the N gene sequences showed that all of the isolates belonged to genotype H1 except 3 isolates, which were genotype A. The nucleotide sequence and predicted amino acid homologies of the 294-genotype H1 strains were 94.7%–100% and 93.3%–100%, respectively. The genotype H1 isolates were divided into 2 clusters, which differed by approximately 2.9% at the nucleotide level. Viruses from both clusters were distributed throughout China with no apparent geographic restriction and multiple co-circulating lineages were present in many provinces. Even though other measles genotypes have been detected in countries that border China, this report shows that genotype H1 is widely distributed throughout the country and that China has a single, endemic genotype. This important baseline data will help to monitor the progress of measles control in China.

## Background

Measles virus (MV), an enveloped virus with a single-stranded, negative sense RNA genome, is a member of the genus *Morbillivirus *within the family *Paramyxoviridae*. MV is highly contagious and causes a disease characterized by high fever, cough, coryza, conjunctivitis and appearance of a maculopapular rash [1]. Despite widespread use of a safe and effective vaccine, it is estimated that MV still causes close to half a million deaths per year and is a major cause of child mortality, mostly in developing countries [2]. However, measles has been eliminated in countries that have maintained high vaccine coverage rates, and four of six WHO regions now have measles elimination goals [3,4].

MV is a monotypic virus, but genetic variability exists among wild type strains [5]. Molecular epidemiological studies have provided an important tool for mapping transmission routes, documenting the elimination of endemic virus strains, and differentiating vaccine from wild-type strains [6-9]. The protocols and nomenclature for genetic characterization of wild-type measles viruses have been standardized by the World Health Organization (WHO) [10]. The WHO currently recognizes 23 genotypes of MV [10-14] based on sequence analysis of the 450 nucleotides coding for the 150 amino acids at the carboxyl-terminus of the nucleoprotein (N) and the coding region of the hemagglutinin (H) gene [10,11]. WHO recommends that genetic analysis of MV isolates should be conducted during all phases of measles control [15].

China, which successfully eradicated wild-type poliomyelitis virus in 1994, now has established a goal for measles elimination by 2012. One of the strategies to achieve elimination includes strengthening measles surveillance. An accelerated measles control program and surveillance activities were initiated in China during 1997 and 1998, respectively. To improve surveillance, a laboratory network was started in 2001, and is currently composed of one national measles laboratory, 31 provincial measles labs and 331 prefecture labs. Measles laboratory surveillance includes serologic confirmation of suspected measles cases and genetic characterization of wild-type viruses.

Analysis of wild-type MV circulating in China during 1993–1995 and 1998–1999 led to the initial identification of a novel genotype, H1 [16,17]. Genotype H1 viruses were isolated in Hunan, Shandong, Hebei, Beijing, Hainan, and Anhui Provinces and were linked to cases detected in the USA in 1997 and 1998 [7]. Epidemiological data from the exported cases suggested that the H1 viruses have a very wide distribution in China including Hong Kong and Guangzhou [7,17]. However, continued sampling of measles virus strains from different locations within China is needed for a more complete understanding of the distribution of genotypes. This report greatly expands our knowledge of wild-type MVs in China. We describe the genetic characterization of nearly 300 wild-type MVs circulating in China between 1995 and 2003. Viral isolates were obtained from 24 of 31 provinces. The results showed widespread distribution of genotype H1 viruses.

## Results and Discussion

**Figure 1:**
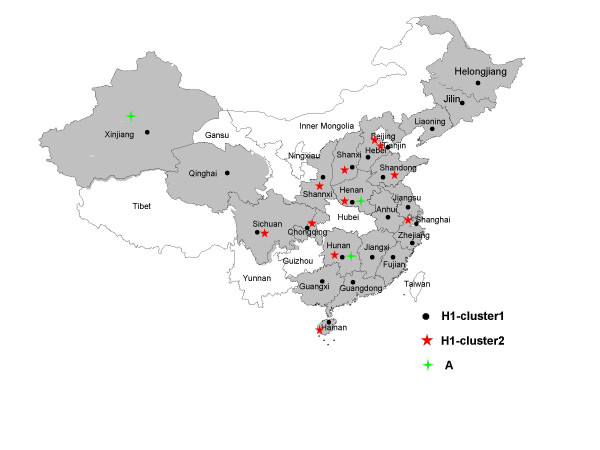
The geographic distribution of Chinese measles isolates from 1995 to 2003. No isolates were received from provinces in white.

**Figure 2:**
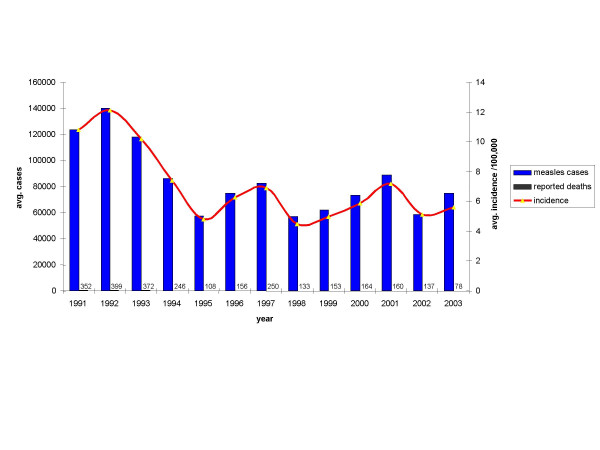
Average number of measles cases (blue bars) and reported death (black bars) and average measles incidence (red line) between 1991 and 2003 in China. Number of reported deaths for each year is indicated above each black bar.

To support further progress in measles control the Ministry of Health of China issued a Plan for Accelerated Measles Control in 1998 and National Measles Surveillance Plan in 2004. The current National Measles Surveillance Plan divides the provinces into 2 groups based on average annual measles incidence [18]. Provinces in Group A have an average measles incidence <6/100,000 and a measles elimination and outbreak prevention goal. Provinces in Group B have an average measles incidence >6/100,000 and a measles control goal. Viral isolates were obtained from 17/18 Group A provinces and 7/13 Group B provinces. The majority of the isolates (84%) were from the Group A provinces because the laboratory network is not as well established in the Group B provinces. Many of the laboratories in Group B provinces lack the necessary equipment and supplies to obtain samples for viral isolation. Table 1 lists the number of isolates obtained from each province in China and the location of the provinces is shown in Figure 1. During 1995–2003, the incidence of measles in China was <8/100,000, with fewer than 250 measles deaths reported each year (Figure 2).

**Table 1:** Number of wild-type measles viruses analyzed between 1995 and 2003 by province. Epidemiologic classification of each province is shown.

Class*	**Province**	**No. of isolates**	**Year of isolates (No.)**	**Genotype**		
				H1		
					
				cluster 1	cluster 2	A
A	Hainan	14	99(1),01(5),03(8)	12	2	0
	Guangdong	7	01(3), 02(4)	7	0	0
	Hunan	9	95(4), 96(1), 01(4)	4	4	1
	Henan	38	99(14),00(12),01(6),02(2),03(4)	28	9	1
	Shandong	23	97(1),99(1),00(5),01(10),02(5),03(1)	21	2	0
	Jiangxi	2	03(2)	2	0	0
	Fujian	1	02(1)	1	0	0
	Anhui	9	98(1),00(2), 01(3),02(3)	9	0	0
	Jiangsu	3	03(3)	3	0	0
	Shanghai	85	00(1),01(14),02(20),03(50)	79	6	0
	Heilongjiang	1	02(1)	1	0	0
	Jilin	9	01(5),02(4)	9	0	0
	Liaoning	11	00(1),01(5),02(4),03(1)	11	0	0
	Innermongolia	0	0	0	0	0
	Shanxi	18	00(3),01(7),02(7),03(1)	13	5	0
	Hebei	1	03(1)	1	0	0
	Tianjin	17	02(13),03(4)	16	1	0
	Beijing	1	00(1)	0	1	0
B	Xinjiang	7	01(1), 02(3),03(3)	6	0	1
	Qinghai	2	00(2)	2	0	0
	Ningxia	0	0	0	0	0
	Gansu	0	0	0	0	0
	Shannxi	9	00(3),01(4),03(2)	7	2	0
	Chongqing	14	00(5),01(4),03(5)	8	6	0
	Tibet	0	0	0	0	0
	Yunnan	0	0	0	0	0
	Guizhou	0	0	0	0	0
	Sichuan	11	02(1),03(10)	10	1	0
	Hubei	0	0	0	0	0
	Zhejiang	3	99(3)	3	0	0
	Guangxi	2	01(2)	2	0	0

	total	297		255	39	3

* See definition of epidemiologic class in the text

**Figure 3:**
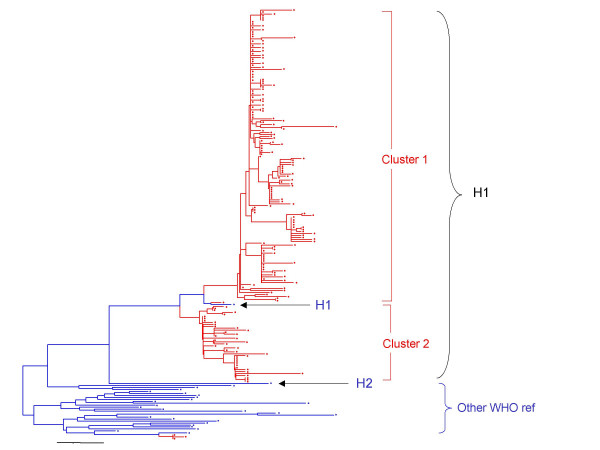
Schematic phylogenetic tree of the N gene sequences of 191 wild-type measles isolates from China compared to the WHO reference sequences for each genotype. For simplicity, strain names are not shown. Sequences from viruses isolated in China from 1995–2003 are indicated by red branches and dots, and WHO reference strains and Shanghai-191 vaccine strain are indicated by blue branches and dots. Positions of the reference strains for genotypes H1 and H2 are indicated by arrows.

**Figure 4:**
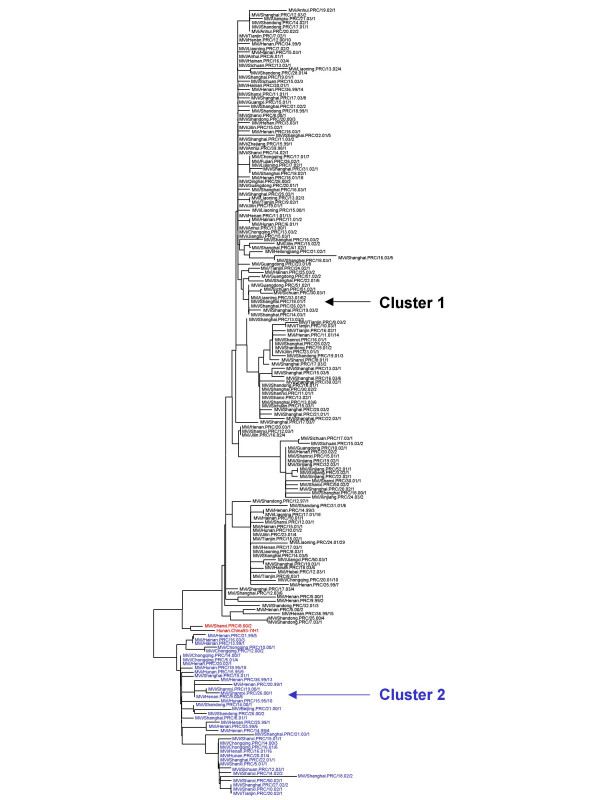
Phylogenetic tree of the N gene sequences of 188 wild-type measles isolates from China in genotype H1. The WHO reference strains and another strain on the intermediate cluster are shown in red. Cluster 1 is shown in black, while cluster 2 is shown in blue. WHO strain name is indicated for each sequence.

The sequences of the 450 nucleotides coding for the COOH-terminus of the nucleoprotein were derived from all of the 297 viral isolates listed in Table 1 and 191 representative sequences were used for phylogenetic analysis. All of the isolates were placed in genotype H1 except 3 isolates, which were in genotype A (Figure 3, 4). The grouping of the sequences within the genotype H1 was supported by bootstrap analysis (data not shown). The ranges of nucleotide sequence and amino acid homologies among the 294 contemporary H1 isolates were 94.7%–100% and 93.3%–100%, respectively.

**Figure 5:**
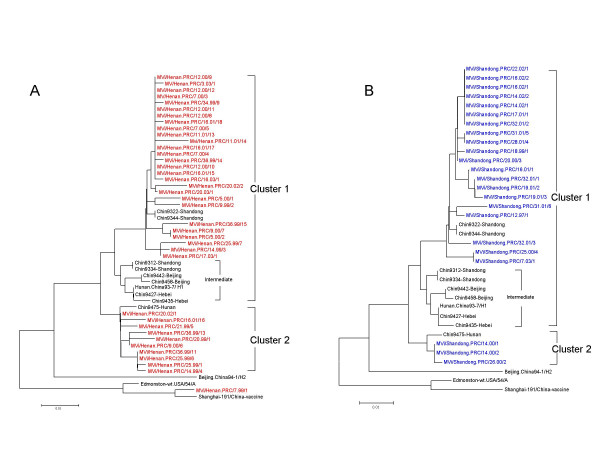
Phylogenetic tree of wild-type measles viruses isolated in Henan (panel A) and Shandong (panel B) provinces between 1995 and 2003. Recent viruses from Henan are shown in red and recent viruses from Shandong are shown in blue. The intermediate cluster containing the reference sequence for genotype H1 and older strains is shown in black. Reference strains for genotypes H2 and A are also shown.

Three of the viral isolates, MVi/Henan.PRC/7.99/1, MVi/Hunan.PRC/15.96/10, MVi/Xinjiang.PRC/24.03/1, belonged to genotype A and the N gene sequences of these viruses were very closely related to the sequence of the vaccine strain used in China, Shanghai-191. Pairwise comparisons of 3 genotype A isolates to Shanghai-191 showed no more than 1.1% nucleotide variation. When these sequences were compared the prototype Edmonston strain, the nucleotide variation was 1.8%–2%. These genotype A viruses also shared several nucleotide substitutions that are unique to the Shanghai-191 strain (Figure 5A) [19]. Fever and rash occur in approximately 5% of measles vaccine recipients within 10–12 days after vaccination [20]. Though immunization histories were not available for these cases, the sequence information shows that these isolates likely represent measles vaccine viruses.

While the phylogenetic analysis clearly showed that the vast majority of the viral isolates were in genotype H1, there were no other obvious patterns. Within this large group of genotype H1 sequences, there was no evidence for geographic or temporal restriction since identical sequences were detected in multiple provinces at the same time and identical sequences were sometimes detected during different years in the same province. The sequence data suggest the presence of sustained chains of transmission in most of provinces, even in some with high immunization coverage rates, such as Shandong and Henan (Figure 5A, 5B). Sustained chains of transmission also were found from 2001 to 2003 in Shanghai City, which may have been due to the influx of a large number of undocumented individuals (floating people).

The phylogenetic tree (Figures 3, 4) shows that the sequences of the genotype H1 viruses from China from 1995–2003 form two major clusters. A third, intermediate cluster, contains sequences of some of the initial sequences reported from China [16] including the reference strain for genotype H1. However, only 1 contemporary sequence, from 2000, groups with this third cluster suggesting that viruses in this lineage may no longer be circulating in China. The amount of nucleotide variation between the 2 large clusters is as high as 2.9%. Within all of the 294 Chinese sequences, the nucleotide variation was up to 5.3% for the 450-nucleotide sequence of the N gene. Therefore, the intra-genotype sequence diversity for genotype H1 is greater than that measured for some other measles genotypes. However, viruses from both major clusters were distributed throughout China without any apparent geographic pattern (Table 1 and Figure 1). Therefore there is no practical reason to separate the genotype H1 viruses into multiple genotypes, at this time, because this would not enhance our ability to describe transmission pathways in China. It is important to note that the genetic heterogeneity of the Chinese measles viruses is not a result of increased mutation rates but is due to the presence of multiple, co-circulating lineages of virus within an endemic genotype.

The initial genetic characterization of wild-type measles viruses from China identified a new genotype H1, which was detected only in China and cases exported to other countries from China [16,21]. A number of genotypes have been detected in the countries that have borders with China including genotypes D4 and D8 in Nepal and India, D4 in Pakistan, G2 in Thailand, H1 in Mongolia, and H2 in Vietnam. However, there are no reports about measles genotypes from the countries that are on China's western borders, but virologic surveillance is improving in these areas [14].

The results of this report greatly expand the scope of the previous work to clearly demonstrate that genotype H1 has a widespread distribution throughout China. The H1 genotype was also detected in large outbreaks in both Japan, and Korea in 2000–2001 [22,23]. Genotypes D3, D5, and D9 were the most frequently detected genotypes in Japan before the outbreaks associated with genotype H1 suggesting that the recent H1 viruses in Japan originated in China. Baseline virologic surveillance had not been conducted in Korea before the large outbreak of genotype H1 in 2000–2001.

Based on the virologic surveillance data, the transmission pattern of genotype H1 viruses in China is consistent with ongoing endemic transmission of multiple lineages of a single, endemic genotype. This type of transmission pattern has also been described for other countries with endemic transmission including Nigeria, Ghana, India, Cambodia, Turkey, Vietnam [17,24-27]. In contrast, in areas, such as the USA, Australia, Canada and the United kingdom where endemic transmission of virus has been interrupted, the number of measles cases is very low and a variety of genotypes are detected, reflecting the multiple sources of imported viruses [7,21,28-30]. Since WPRO, including China, has recently initiated a program to eliminate measles by 2012, this report provides important baseline virologic information for China. Hopefully, virologic surveillance in China will be able to document the interruption of transmission of genotype H1 viruses and, in conjunction with epidemiological surveillance, will help to identify the sources of imported virus.

## Conclusion

This study reports virologic surveillance data obtained in China during a period when measles control activities were greatly accelerated. The results confirmed that genotype H1 is the endemic genotype throughout China. The virologic data were consistent with endemic measles in that multiple chains of transmission were evident. The H1 viruses were very diverse and formed two major clusters, which were distributed throughout China with no apparent geographic restriction. This important baseline data will contribute to the development of improved measles control programs in China.

## Methods

### Specimens collection and virus isolation

Throat swab and urine samples were obtained from serologically confirmed measles cases. Clinical specimens were inoculated onto B95a cells [31], and the cells were observed for cytopathic effect (CPE). Inoculated cells were blind-passaged up to three times before being discarded. Cells were harvested when the CPE was maximal. Virus isolations were performed by 24 provincial laboratories in China and the viral isolates were shipped to the National Measles Laboratory, in Beijing for genetic analysis.

### RNA Extraction and RT-PCR

Viral RNA was extracted from infected cell lysates using Trizol reagent according to the manufacturer's directions. RNA pellets were dried and resuspended in 50 μl of sterile distilled water and stored at -70 C until amplification by RT-PCR. RT-PCR was performed using previously described methods [6,20]. Primers MV63 (5'CCT CGG CCT CTC GCA CCT AGT 3') and MV60 primers (5'GCT ATG CCA TGG GAG TAG GAG TGG 3') were used to amplify a 676 bp fragment of the N gene including the 450 bp fragment recommended for genotyping.

### Sequence analysis

The sequences of the PCR products were derived by automated sequencing with primers MV60 and MV63 and the BigDye terminator v2.0 chemistry using reaction conditions that were recommended by the manufacturer (ABI 373, ABI 3100, Perkin Elmer-Applied Biosystems). Sequence proof reading and editing was conducted with Sequencer™ (Gene Codes Corporation). Sequence data were analyzed by using version 7.0 of Bioedit and phylogenetic analyses were performed using Bioedit and Mega ver3.1. The robustness of the groupings was assessed using bootstrap resampling of 1000 replicates and the trees were visualized with Mega programs. 191 representative nucleotide sequences were deposited in GenBank under accession numbers DQ356683–DQ356873.

## List of abbreviations

MV: Measles virus

RT-PCR: reverse transcriptase polymerase chain reaction

H: Hemagglutinin

N: Nucleoprotein

WHO: World Health Organization

## Competing interests

The author(s) declare that they have no competing interests.

## Authors' contributions

YZ, PAR, DF, WJB, GDL, WBX prepared manuscript. WBX designed the study and organized the coordination. YZ performed sequence and data analysis. YZ, ZZ, YQL, YXJ, STX performed RT-PCR and sequence analysis. XHJ, JYH, JGW, WT, ZYZ, CYL, CYW, TZW, LZ, HT, HL, CFZ, YM, CYL, JLH, JT, YM, PL, RHG, WKH, JHZ, GYL, HZ, XGY, XLY, JLZ, YYL, SDZ, ZMB, WL, XHY, YJM, YL collected specimens and performed virus isolation, viral identification and measles IgM assays for case confirmation. All authors read and approved the final manuscript.

Current address for Yeqiang Li is Towson University. 8000 York Road, Towson, Maryland 21252.
